# Optogenetic Stimulation of Cortex to Map Evoked Whisker Movements in Awake Head-Restrained Mice

**DOI:** 10.1016/j.neuroscience.2017.04.004

**Published:** 2018-01-01

**Authors:** Matthieu Auffret, Veronica L. Ravano, Giulia M.C. Rossi, Nicolas Hankov, Merissa F.A. Petersen, Carl C.H. Petersen

**Affiliations:** Laboratory of Sensory Processing, Brain Mind Institute, Faculty of Life Sciences, École Polytechnique Fédérale de Lausanne (EPFL), Switzerland

**Keywords:** ChR2, channelrhodopsin-2, FFT, Fourier transform, PtA, parietal association cortex, whisker motor control, motor cortex, sensory cortex, barrel cortex, optogenetics, cortical motor map

## Abstract

Whisker movements are used by rodents to touch objects in order to extract spatial and textural tactile information about their immediate surroundings. To understand the mechanisms of such active sensorimotor processing it is important to investigate whisker motor control. The activity of neurons in the neocortex affects whisker movements, but many aspects of the organization of cortical whisker motor control remain unknown. Here, we filmed whisker movements evoked by sequential optogenetic stimulation of different locations across the left dorsal sensorimotor cortex of awake head-restrained mice. Whisker movements were evoked by optogenetic stimulation of many regions in the dorsal sensorimotor cortex. Optogenetic stimulation of whisker sensory barrel cortex evoked retraction of the contralateral whisker after a short latency, and a delayed rhythmic protraction of the ipsilateral whisker. Optogenetic stimulation of frontal cortex evoked rhythmic bilateral whisker protraction with a longer latency compared to stimulation of sensory cortex. Compared to frontal cortex stimulation, larger amplitude bilateral rhythmic whisking in a less protracted position was evoked at a similar latency by stimulating a cortical region posterior to Bregma and close to the midline. These data suggest that whisker motor control might be broadly distributed across the dorsal mouse sensorimotor cortex. Future experiments must investigate the complex neuronal circuits connecting specific cell-types in various cortical regions with the whisker motor neurons located in the facial nucleus.

## Highlights

• Optogenetic whisker motor mapping in awake head-restrained mice.
• Stimulation of whisker sensory cortex evokes contralateral whisker retraction.
• Stimulation of frontal cortex evokes bilateral rhythmic whisker protraction.
• Stimulation of posteriomedial cortex evokes bilateral rhythmic whisker protraction.

## Introduction

Rodents use their array of mystacial whiskers to obtain tactile information about their immediate facial surroundings (Brecht, 2007, Petersen, 2007, Diamond et al., 2008, Bosman et al., 2011, Feldmeyer et al., 2013). During active exploration rodents typically move their whiskers back and forth at high frequencies (∼10 Hz) to sample the space around their snouts (Welker, 1964). As a whisker contacts an object, it bends and the resulting force is transduced into action potential firing in the primary sensory neurons of the trigeminal ganglion, which innervate the whisker follicles. Sensory information can therefore be actively acquired by rodents through self-generated movements causing whisker–object contact. Whisker sensory information flowing into the rodent brain is thus in part determined by whisker motor control. In order to understand whisker sensory perception, we therefore also need to investigate the mechanisms underlying the control of whisker movements.

Movements are controlled by complex neuronal circuits, including an important influence by the neocortex. Pioneering experiments in dogs (Fritsch and Hitzig, 1870), monkeys (Ferrier, 1874, Sherrington, 1906) and man (Penfield and Boldrey, 1937) revealed important organizing principles of mammalian cortical motor control. Electrical stimulation of different cortical regions evoked different movements, with the most important region, the primary motor cortex (M1), being located in the frontal cortex, anterior to the central sulcus. Stimulation of different sites in M1 evoked movements, which appear to mirror the somatotopic organization of sensory cortex.

Early experiments in rodents suggested that movements could be evoked by stimulating many different regions of the neocortex (Hall and Lindholm, 1974, Donoghue and Wise, 1982, Gioanni and Lamarche, 1985, Neafsey et al., 1986). Motor maps revealed forelimb and hindlimb motor representations bordering with their sensory representations, whereas head, whisker, and eye movements were found to be preferentially evoked by stimulation of more anterior and medial locations (Woolsey, 1958, Hall and Lindholm, 1974, Donoghue and Wise, 1982, Neafsey et al., 1986, Miyashita et al., 1994, Brecht et al., 2004).

A number of studies have specifically investigated the effects of cortical stimulation upon whisker movements finding diverse results. In awake head-restrained mice, stimulation of the primary somatosensory whisker barrel cortex (wS1) has been proposed to evoke retraction of the contralateral whisker, whereas the direct effect of stimulation of a frontal region wM1, which is strongly innervated by wS1, is proposed to drive rhythmic whisker protraction (Matyas et al., 2010, Petersen, 2014, Sreenivasan et al., 2015, Sreenivasan et al., 2016). In contrast, recent work in freely moving rats suggested that neuronal activity in an apparently analogous region to wM1, may suppress contralateral whisking (Ebbesen et al., 2017). A further study in lightly anesthetized rats, proposed that a rhythmic whisking region be located in a more posterior and medial cortical region (Haiss and Schwarz, 2005). The diverse results in terms of whisker movements evoked by stimulating different regions of the rodent cortex may result from differences in species, stimulation methods, or behavioral context. Further experiments are therefore necessary in order to understand the organization of cortical whisker motor control. Here, we use optogenetic stimulation of cortex in transgenic mice expressing channelrhodopsin-2 (ChR2) (Nagel et al., 2003, Boyden et al., 2005, Arenkiel et al., 2007, Ayling et al., 2009, Hira et al., 2009, Matyas et al., 2010, Harrison et al., 2012) to begin to map the whisker movements evoked by the same light stimulus applied to many different regions of the dorsal cortex. Our results indicate that whisker movements can be evoked by stimulating many cortical regions, with short latency retraction of contralateral whiskers being evoked from wS1, and rhythmic bilateral whisker protraction being evoked by stimulation of other cortical areas including a frontal and a more posterior midline cortical region.

## Experimental procedures

### Animal preparation and surgery

All experiments were carried out in accordance with protocols approved by the Swiss Federal Veterinary Office. In this study we used four transgenic mice (two male and two female, age ∼3 months) expressing ChR2 under the Thy1 promoter: mouse strain name B6.Cg-Tg(Thy1-COP4/EYFP)18Gfng/J, JAX mouse number 07612, RRID: IMSR_JAX:007612 (Arenkiel et al., 2007). Mice were anesthetized under deep isoflurane and a metal head-holder implanted. A relatively transparent view of the left dorsal cortex was prepared following previously published methods (Guo et al., 2014b). In brief, the skull was covered with a thin layer of cyanoacrylic glue, and then a thick layer of transparent dental acrylic cement (Jet Repair Acrylic) was applied. Three days after the implantation the cement was polished using a polishing kit. In a final step, the polished cement was covered with nail polish, to make the surface of the skull even and transparent. All whiskers were trimmed except the C2 whiskers on either side.

### Optogenetic mapping of evoked whisker movements

**Fig. 1:**
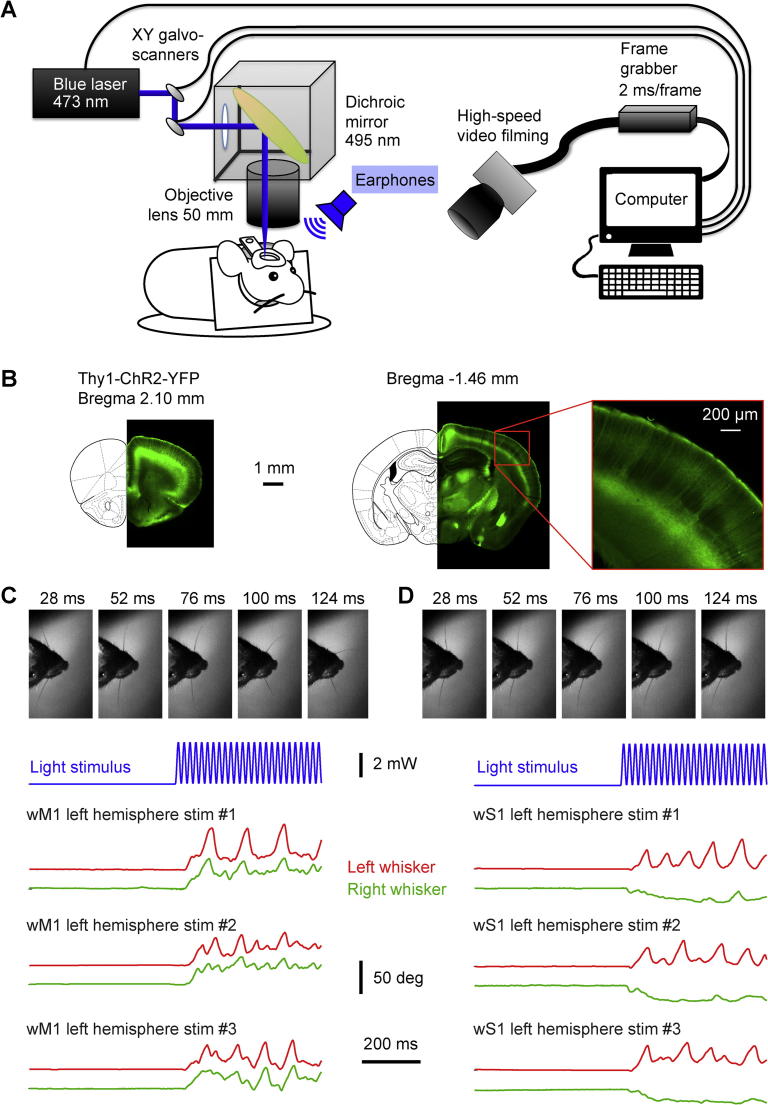
Experimental setup for optogenetic whisker motor mapping. (A) The left hemisphere of Thy1-ChR2-YFP mice was stimulated by 473 nm blue laser light with a 50-Hz sine wave modulation. The beam was directed by two scanning galvanometer mirrors onto a dichroic mirror that reflected the blue light to the surface of the skull through a 50-mm focal length camera lens to focus the beam on specific locations of the mouse cortex. A high-speed video camera filmed the C2 whiskers of both left and right sides at 500 Hz. Blue ambient light indirectly illuminated the background and masked the laser light. White noise was played to cover noise from galvanometer mirrors and any ambient noise. Laser stimulation, galvanometer mirrors and high-speed video filming were controlled by a computer. (B) YFP fluorescence in fixed coronal slices of a Thy1-ChR2-YFP mouse imaged at 4× magnification at two different anterior–posterior locations, ∼2.10 mm frontal to Bregma (close to wM2, left image) and ∼1.48 mm posterior to Bregma (center image) where we observed the barrel cortex structure of wS1. Schematic drawings were adapted from Paxinos and Franklin (2001). A zoomed-in version of the barrel cortex was acquired with a 10× magnification lens (right image). Layer 5 pyramidal neurons and their dendritic arborizations extending to superficial layers were observed. (C and D) Example of raw movie images of the mouse MA034 at five different times (28, 52, 76, 100 and 124 ms after stimulus onset), during wM1 (left) and wS1 (right) stimulation trial #1 of left hemisphere. The temporal pattern of the laser light stimulus delivered to the mouse cortex at wM1 and wS1 localization is shown in blue. Below are three example trials of left and right whisker angles tracked from the high-speed movies for both wM1 (left) and wS1 (right) stimulation. wM1 stimulation drove protraction of both whiskers, whereas wS1 stimulation drove protraction of the ipsilateral whisker and retraction of the contralateral whisker.

**Fig. 2:**
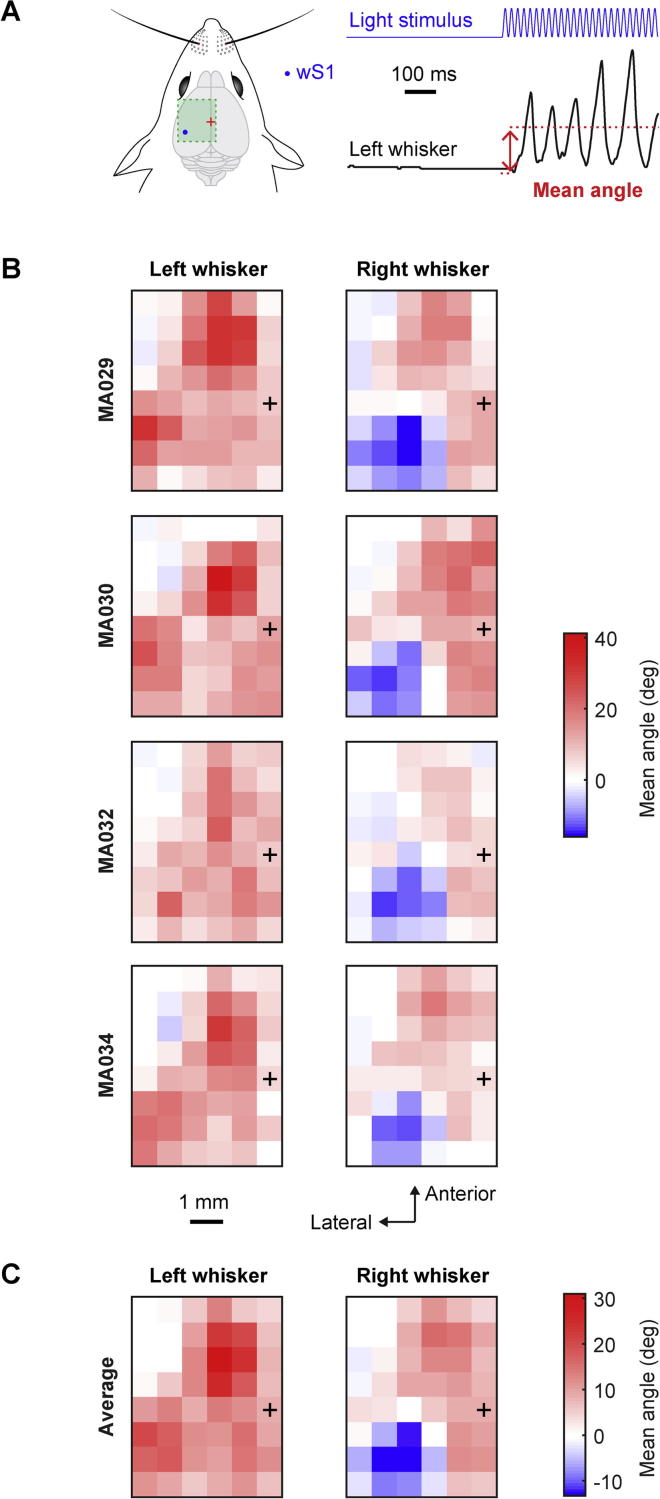
Mapping of the average change in whisker angle evoked by optogenetic stimulation. (A) Schematic drawing of wS1 stimulation (left) and an example of the left whisker angle (right) showing how the mean angle was computed: the difference in mean whisker angle during the 500 ms of optogenetic stimulation compared to the mean whisker angle during the two frames (4 ms) before the stimulus onset. (B) The mean change in angle for the left C2 whisker and right C2 whisker of each mouse represented on a 2D color-coded map corresponding to each stimulation coordinate on the left hemisphere. The amplitude of the mean change in angle corresponds to the median of all the trials where the mouse did not whisk before the stimulus (whisker angle standard deviation less than 1° for 200 ms before the stimulation). Positive values reflect a protraction of the whisker and negative values indicate retraction. Bregma position is represented by a black cross. (C) Average over the four mice of the mean angle positions for left and right C2 whiskers relative to the stimulation coordinates on the left hemisphere. There was a large protraction of both whiskers when wM1/wM2 was stimulated. Protraction of the ipsilateral whisker and a retraction of the contralateral whisker was evoked when wS1 was stimulated.

**Fig. 3:**
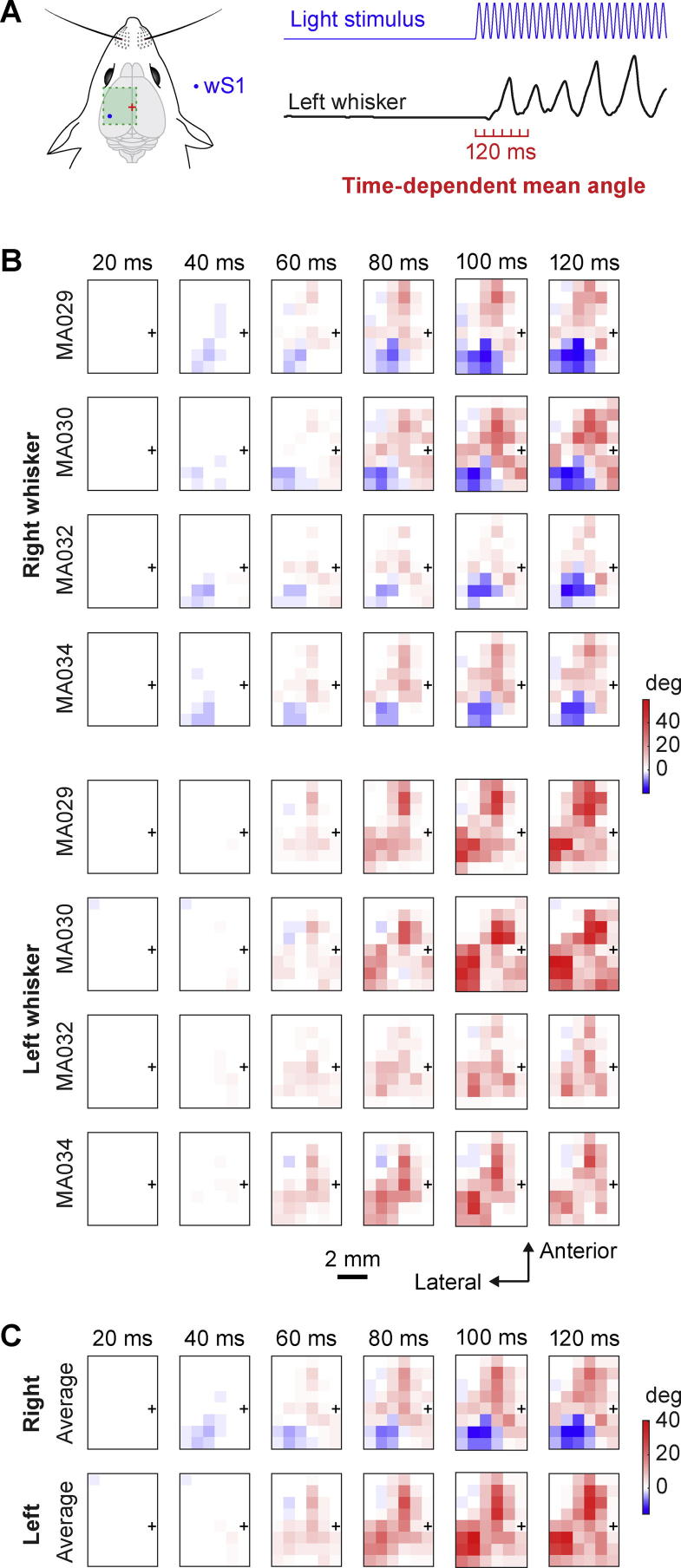
Mapping of the time-dependent change in whisker angle evoked by optogenetic stimulation. (A) Schematic drawing of wS1 stimulation (left) and an example of the left whisker angle (right) showing how the time-dependent whisker angle was computed: the mean change in whisker angle during six consecutive time bins (0–20, 20–40, 40–60, 60–80, 80–100, and 100–120 ms relative to stimulation onset) from the mean whisker position during the 4 ms before the stimulus onset. (B) Mean whisker angle during the six 20-ms time bins for the left C2 whisker and right C2 whisker of each mouse represented on a 2D color-coded map corresponding to each stimulation coordinate of the left hemisphere. The amplitude of the mean angles reported corresponds to the median of all the trials where the mouse did not whisk before the stimulus (whisker angle standard deviation less than 1° for 200 ms before the stimulation). Positive values reflect a protraction of the whisker and negative values indicate retraction. Bregma position is represented by a black cross. (C) Average over the four mice of the time-dependent mean angle positions for left and right C2 whiskers relative to the stimulation coordinates of the left hemisphere. The first movement evoked was in the 20–40-ms time-window, and was a retraction of the contralateral whisker when wS1 cortex was stimulated. In the 40–60-ms time-window, a protraction of both whiskers was evoked when wM1/wM2 and PtA were stimulated.

**Fig. 4:**
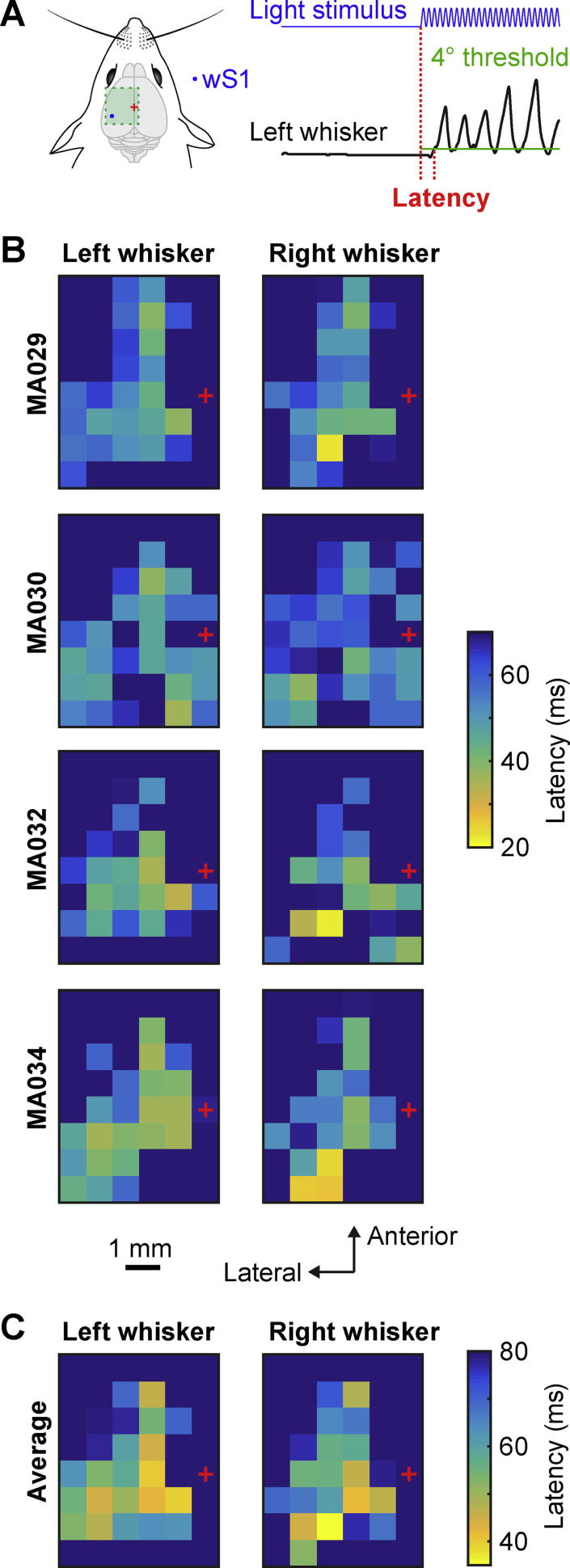
Latency maps of whisker movements evoked by optogenetic stimulation. (A) Schematic drawing of wS1 stimulation (left) and an example of the left whisker angle (right) showing how the latency was computed: time relative to stimulation onset when the whisker moved more than 4° compared to its initial position. (B) Latencies for the left C2 whisker and right C2 whisker of each mouse represented on a 2D color-coded map corresponding to each stimulation coordinate of the left hemisphere. Trials in which the mouse did not move its whisker by more than 4° were not included in the latency analysis. The value of latencies reported corresponds to the median of all the trials where the mouse did not whisk before the stimulus (whisker angle standard deviation below 1° for 200 ms before the stimulation). Bregma position is represented by a red cross. (C) Average over the four mice of the latencies for left and right C2 whiskers relative to the stimulation coordinates on the left hemisphere. The shortest latencies were for contralateral whisker retraction when wS1 was stimulated.

**Fig. 5:**
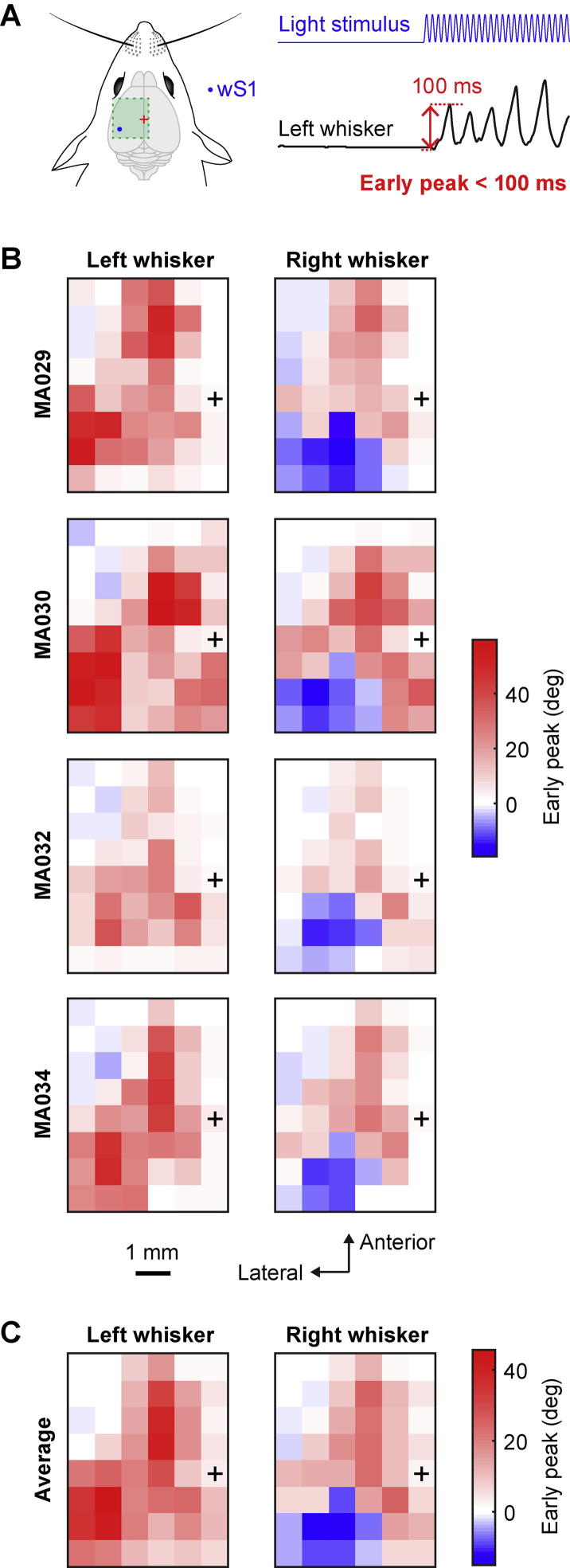
Maps of the early peak whisker movement evoked by optogenetic stimulation. (A) Schematic drawing of wS1 stimulation (left) and an example of the left whisker angle (right) showing how the early peak parameter was computed: the maximum (for protraction) or minimum (for retraction) change in whisker angle during the first 100 ms after the stimulus onset. (B) Early peak whisker movement for the left C2 whisker and right C2 whisker of each mouse represented on a 2D color-coded map corresponding to each stimulation coordinate on the left hemisphere. The value of the early peak movement reported corresponds to the median of all the trials where the mouse did not whisk before the stimulus (whisker angle standard deviation below 1° for 200 ms before the stimulation). Positive values reflect a protraction of the whisker and negative values indicate retraction. Bregma position is represented by a black cross. (C) Average over the four mice of the early peak change in whisker angle for left and right C2 whiskers relative to the stimulation coordinates on the left hemisphere. The largest early whisker protraction was evoked by stimulating wM1/wM2. Stimulating wS1 evoked a large early protraction of the ipsilateral whisker and a large early retraction of the contralateral whisker.

**Fig. 6:**
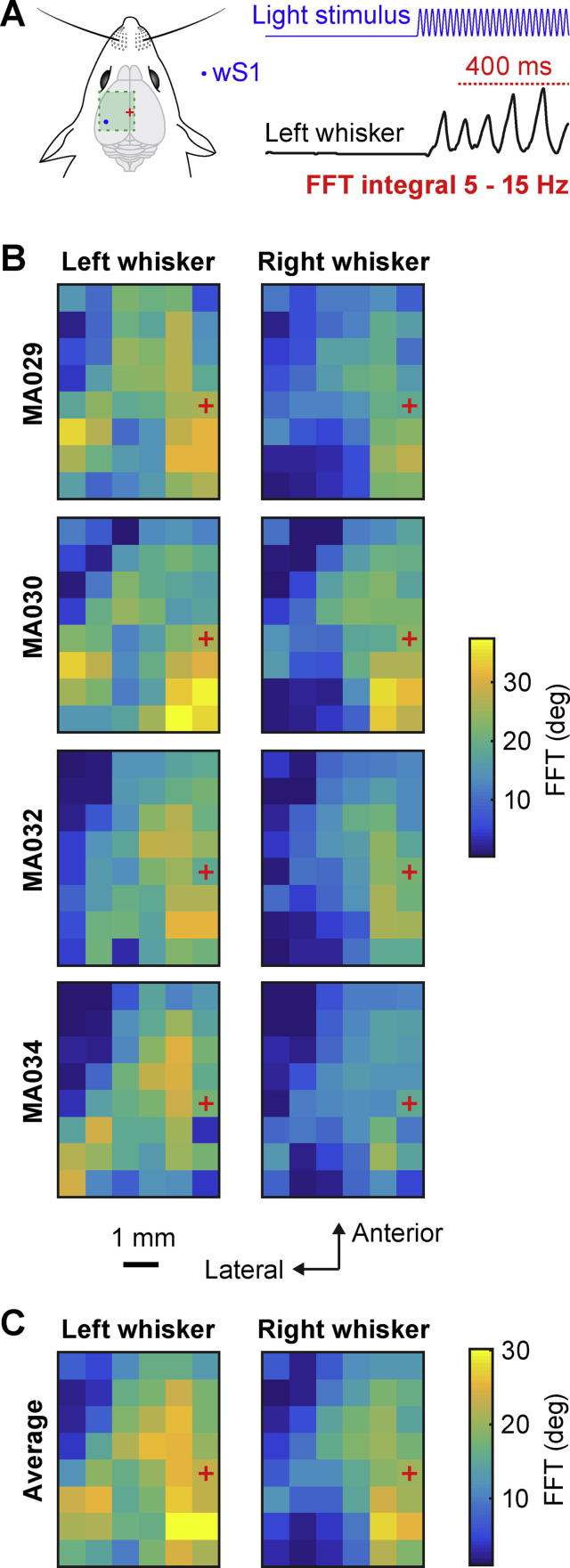
Maps of the amplitude of whisker movements in the 5–15-Hz frequency range evoked by optogenetic stimulation. (A) Schematic drawing of wS1 stimulation (left) and an example of the left whisker angle (right) showing how the fast Fourier transform (FFT) was computed: the integral from 5 Hz to 15 Hz of the FFT during the last 400 ms of the laser light stimulation. (B) FFT values for the left C2 whisker and right C2 whisker of each mouse represented on a 2D color-coded map corresponding to each stimulation coordinate on the left hemisphere. The value of FFT reported corresponds to the median of all the trials where the mouse did not whisk before the stimulus (whisker angle standard deviation less than 1° for 200 ms before the stimulation). Bregma position is represented by a red cross. (C) Average over the four mice of the FFT for left and right C2 whiskers relative to the stimulation coordinates on the left hemisphere. The largest 5–15-Hz whisker movements were evoked by stimulating the PtA region (posterior to Bregma close to the midline).

**Fig. 7:**
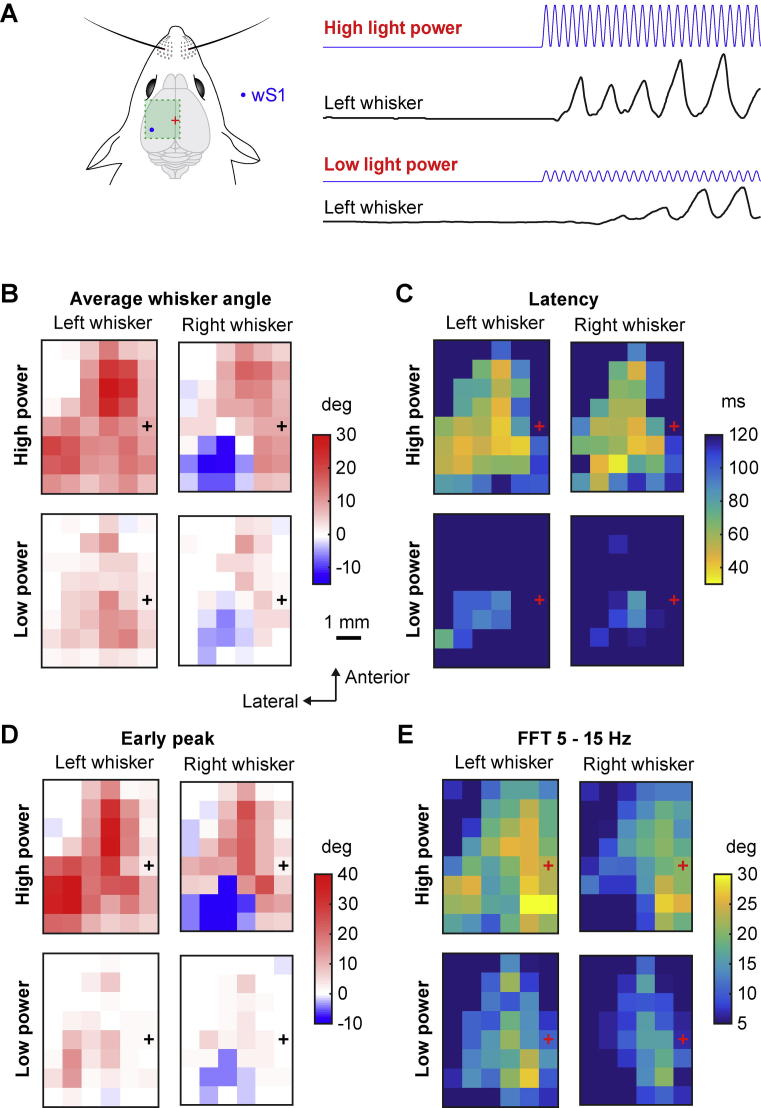
Motor maps evoked by different blue light intensities. (A) Schematic drawing of wS1 stimulation (left) and examples of the left whisker angle (right) for two wS1 stimulation trials with either high laser light power (mean power 1.75 mW) or low laser light power (mean power 0.36 mW). (B) Comparison of the averaged left and right whisker mean angle positions for the high and low power trials. The whiskers protracted/retracted less for low power stimulation compared to the high power, but the motor map pattern stayed relatively comparable. The high power map is the same as shown in Fig.2C. (C) Comparison of the averaged left and right whisker movement latencies for the high and low light power trials. The latencies of the whisker movements increased in low power stimulation compared to high power, but the smallest latencies observed were still located around wS1. The high power map is the same data as shown in Fig.4C. (D) Comparison of the averaged left and right early peak whisker movement amplitudes for the high and low light power trials during the first 100 ms after stimulus onset. The amplitudes of the early movements were reduced with low power but the largest protractions observed for both conditions were located in wM1/wM2 for the two whiskers and wS1 for the ipsilateral whisker, and the largest retraction was still observed around wS1 for the contralateral whisker. The high power map is the same as shown in Fig.5C. (E) Comparison of the averaged left and right whisker 5–15 Hz FFT for the high and low light power trials during the last 400 ms of the stimulation. The whisking amplitudes were reduced for low power stimulation compared to high power stimulation, but the largest 5–15-Hz FFT values for both conditions were localized in the PtA region posterior to Bregma close the midline. The high power map is the same as shown in Fig.6C.

**Fig. 8:**
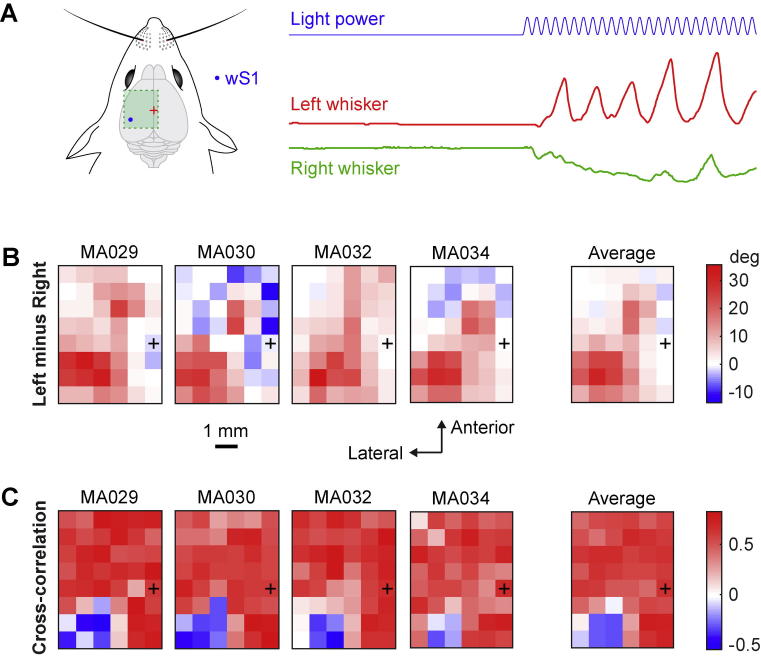
Correlations and differences in the ipsilateral and contralateral whisker movements evoked by optogenetic stimulation. (A) Schematic drawing of wS1 stimulation (left) and an example of the corresponding left and right whisker angles (right). (B) Difference between the left whisker angle and right whisker angle (left minus right) reported on a 2D color-coded map for each mouse (left images) and averaged across the four mice (right image). The left whisker usually protracted more than the right whisker when the left hemisphere was stimulated. (C) Cross-correlations of the left and right whiskers positions during the stimulation were high in almost all cortical areas except for stimulation of wS1, where there was an anti-correlation of the two whiskers.

**Fig. 9:**
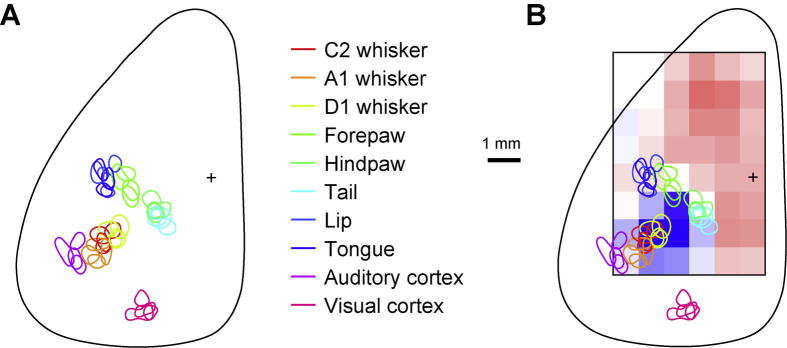
Whisker motor maps in the context of sensory maps. (A) Sensory map obtained with intrinsic optical imaging. Right C2, A1 and D1 whiskers, right forepaw, right hindpaw, tail, lip and tongue were deflected at a frequency of 10 Hz using a mechanical stimulator. A train of click sounds was used to deliver auditory stimuli. Light flashes pointed toward the right eye were used to deliver visual stimuli. The color-coded contours indicate the region of maximal evoked activity in each mouse. (B) Overlay of the sensory map obtained in panel A with the motor map of the right whisker mean angle positions (as shown in Fig.2C). There was a good overlap between the primary sensory whisker cortex (wS1) and the region where stimulation evoked a large retraction of the contralateral whisker.

Mice were adapted to head-restraint through daily training sessions (Crochet and Petersen, 2006). The first head-fixation session was brief (∼15 min), and over the next days the duration of head-restraint was gradually extended to one hour. After adaptation to head-restraint, optogenetic stimuli were applied to different regions of the left cortex, while left and right C2 whiskers were filmed at 500 Hz illuminated with blue light below the mouse to show silhouette and whiskers (Fig. 1). Each trial lasted 1 s with 500 ms of a prestimulus baseline period followed by 500 ms of optogenetic stimulation. The minimal intertrial interval was 5 s. Auditory white noise was constantly played through earphones near to the ears of the mice to mask the noise of the galvanometer mirrors and any ambient noise. The optogenetic stimulus consisted of a blue light spot of ∼500-µm diameter, which varied in intensity with a 50-Hz sine wave modulation, with a peak power of 3.49 mW and mean power of 1.75 mW (Fig. 1, Fig. 2, Fig. 3, Fig. 4, Fig. 5, Fig. 6, Fig. 7, Fig. 8, Fig. 9). In some experiments we used a lower light power with a peak power of 0.72 mW and mean power of 0.36 mW (Fig. 7). The blue light was generated by a 473-nm fiber-coupled laser (Thorlabs, Newton, New Jersey, USA), focused onto the mouse cortex through a 50-mm focal length camera lens (Nikon, Tokyo, Japan), and directed to specific locations on the mouse cortex using 2D scanning galvanometer mirrors (Thorlabs, Newton, New Jersey, USA) controlled by a computer via a digital-to-analog converter (National Instruments, Austin, Texas, USA) (Guo et al., 2014a). A photostimulation grid of 6 × 8 pixels covering an area of 3.9 × 5.0 mm over the left hemisphere was aligned to Bregma, and each point was stimulated in a random order. The whole grid was covered before repeating the stimulus protocol, with each mapping sequence lasting ∼10 min. Altogether, each coordinate was stimulated 12 times at the high laser power and 6 times at the low laser power. For each mouse, motor mapping was conducted across 2 or 3 days in three sessions, each with six repetitions covering the entire stimulus grid.

### Intrinsic optical imaging to map sensory-evoked activity

After allowing the whiskers to regrow for several weeks, we carried out intrinsic optical imaging experiments to map sensory representations. Mice were lightly anesthetized with ∼0.5% isoflurane. The body temperature of the mouse was maintained at 37 °C by a heating pad. A first image of the cortical surface was acquired with 530-nm green LED light in order to locate Bregma and blood vessels. For functional imaging, the illumination was changed to 625-nm red LED light. Different body parts were sequentially mechanically stimulated by a glass capillary attached to a piezo-bender. Three different right whiskers were stimulated (A1, C2 and D1) to assess the whisker somatotopic organization for each individual mouse. The whisker was inserted into the glass tube and was stimulated at 10 Hz for 4 s. The right forepaw, the right hindpaw, the tail, the lip and the tongue were similarly stimulated by tapping the body part with the same piezo system at 10 Hz for 4 s. Auditory stimuli were delivered by click sound pulses at 10 Hz for 4 s. Visual stimuli to the right eye were delivered by flashing a blue LED at 10 Hz for 4 s. Each trial consisted of a 4-s baseline period, followed by 4 s of stimulation, and then 2 s poststimulus. The total trial duration was 10 s and the intertrial interval was 4 s. Images were acquired at 10 Hz with 8.7 × 8.7-mm field of view and a detector of 1024 × 1024 pixels (Photon Focus, Lachen, Switzerland, MV-D1024E-40). Stimulus delivery and image processing were carried out using custom written routines in Matlab (Mathworks, Natick, Massachusetts, USA). For each stimulus, the fractional change in reflected light was computed across an average of 20 trials, and aligned to the location of Bregma.

### Data analysis

Whisker angle was quantified using semi-automated custom-written routines in IgorPro (Wavemetrics, Lake Oswego, Oregon, USA). In a small fraction (3.6%) of trials we were not able to track the whisker angle. Further data analysis was conducted in Matlab. Only trials in which the mouse was not moving its whiskers (<1° standard deviation in whisker angle during the 100 frames, i.e. 200 ms, before the stimulation) were included in our analyses. Using this analysis criterion, 32.1% of the remaining trials were rejected because of prestimulus whisker movement. All numbers in the text are presented as mean ± standard deviation for *n* = 4 mice.

Mean evoked change in whisker angle (Fig. 2) for both right and left C2 whiskers was computed for each trial as the difference in the mean whisker angle during the 500 ms of optogenetic stimulation compared to the mean whisker angle during the 4 ms immediately before optogenetic stimulation. Positive values indicate whisker protraction and negative values whisker retraction. The median value was color-coded in the maps across trials for each mouse (Fig.2B), and then averaged across the four mice (Fig.2C).

Time-dependent mean evoked change in whisker angle (Fig. 3) for both right and left C2 whiskers was computed for each trial as the difference in the mean whisker angle compared to the mean whisker angle during 4 ms immediately before optogenetic stimulation subdivided in 20-ms time-bins over the first 120 ms of stimulation, leading to 6 time-dependent whisker motor maps. The median value was color-coded in the maps across each mouse (Fig.3B), and as the average of these maps across the four mice (Fig.3C).

The latency for evoking whisker movements (Fig. 4) for both right and left C2 whiskers was computed for each trial as the time corresponding to when the whisker angle changed more than ±4° compared to the whisker angle during the 4 ms immediately before optogenetic stimulation. If the whisker did not change angle by more than 4° during the optogenetic stimulation then the trial was not included in the latency analysis (7.9% of trials did not pass threshold). The median value of the latency was color-coded in the maps across each mouse (Fig.4B), and then averaged across the four mice (Fig.4C).

The peak amplitude of early changes in whisker angle within the first 100 ms of optogenetic stimulation (Fig. 5) for both right and left C2 whiskers was computed for each trial as the maximum change in whisker angle (considering both positive values for protraction and negative values for retraction) compared to the whisker angle during 4 ms immediately before optogenetic stimulation. The median value of the early phase peak amplitude was color-coded in the maps across each mouse (Fig.5B), and then averaged across the four mice (Fig.5C).

The amplitude of whisker movements occurring within the frequency range of 5–15 Hz (Fig. 6) for both right and left C2 whiskers was computed for each trial as the integral between 5 Hz and 15 Hz of the fast Fourier transform (FFT) of whisker angle (with the mean value subtracted) during the last 400 ms of the optogenetic stimulation (from 100 ms to 500 ms after the stimulus onset). The median value of the 5–15-Hz FFT integral across trials was color-coded in the maps for each mouse (Fig.6B), and then averaged across the four mice (Fig.6C).

In order to assess the influence of the laser power on the evoked whisker movements of both right and left C2 whiskers, we repeated the same analysis procedures described above to compute the mean angle, the latency, the early peak and the FFT for the lower laser light power. The average value across mice of the mean angle (Fig.7B), the latency (Fig.7C), the early peak (Fig.7D) and the 5–15-Hz FFT (Fig.7E) was color-coded in the maps for high and low laser light power.

The difference of mean change in angle between left and right C2 whiskers was computed for each trial as the difference in the mean angle of the left whisker minus the mean angle of the right whisker during the 500 ms of optogenetic stimulation. The median value was color-coded in the maps across trials for each mouse, and then averaged across all mice (Fig.8B). The cross-correlation between right and left C2 whiskers was computed by taking the amplitude of the cross-correlation between the normalized right whisker trace during the 500 ms of optogenetic stimulation (the whisker trace with the mean value subtracted is divided by its standard deviation) and the normalized left whisker trace at zero time lag. The median value was color-coded in the maps across trials for each mouse, and then averaged across all mice (Fig.8C).

The sensory maps for each mouse were computed by taking the contours at near minimal values of the smoothed intrinsic signal image for each body part. The primary sensory cortex region of each stimulated body part was color-coded, aligned to Bregma, and superimposed (Fig.9A). The sensory maps aligned to Bregma were overlaid with the averaged mean angle of the right C2 whisker motor map across all mice, as shown in Figs. 2C, 9B).

All whisker angle data in Matlab, Python and Excel formats together with the location and timing of optogenetic stimulation are available in the Petersen-lab-data community hosted at https://zenodo.org together with the Matlab analysis code used to generate the results (https://doi.org/10.5281/zenodo.437933).

## Results

### Optogenetic stimulation of whisker movement

A computer-controlled 2D scanning galvanometer mirror directed a blue laser light spot (∼500-µm diameter) onto different regions of the left dorsal sensorimotor cortex of awake head-restrained mice while we filmed left and right C2 whisker movements at 500 Hz (Fig.1A). We used Thy1-ChR2-YFP line 18 mice (Arenkiel et al., 2007), which express ChR2 at high levels in many brain regions including prominent expression in layer 5 pyramidal neurons of the neocortex (Fig.1B). Each trial consisted of an initial 500 ms prestimulus period, followed by 500 ms of optogenetic stimulation during which the blue light was pulsed at 50 Hz with a mean power of 1.75 mW. The minimal intertrial interval was 5 s. Whisker movements of different latencies, amplitudes, directions and rhythmicity were evoked from different cortical regions. Stimulation of some cortical regions reliably evoked bilateral rhythmic whisking (Fig.1C), whereas stimulation of other cortical regions reliably evoked contralateral whisker retraction together with ipsilateral rhythmic whisking (Fig.1D). Only trials in which the mouse was not moving its whiskers during the baseline period were included in our analyses (less than 1° of whisker angle standard deviation in the 200 ms preceding the optogenetic stimulus).

### Mapping the average change in whisker angle

We first computed the average change in whisker angle for left and right whiskers evoked by stimulating different cortical regions, by subtracting the mean whisker angle during the 500 ms of optogenetic stimulation from the prestimulus angle (Fig.2A). For each stimulated cortical region in each of the mice, we computed the median change in whisker angle across individual trials and color-coded the result with red colors indicating protraction, blue colors indicating retraction, and white if the mean whisker angle remained unchanged during the stimulation period (Fig.2B). Whisker movements were evoked by the stimulation of many different cortical locations. In general, the stimulation of nearby cortical locations evoked similar movements. Visual inspection of the whisker motor maps suggested three regions containing separate hot-spots for evoking whisker movement.

In each mouse, we found that stimulation of a posteriolateral region evoked retraction of the contralateral whisker and protraction of the ipsilateral whisker. The stimulus site evoking the largest retraction of the contralateral whisker was located at 2.7 ± 0.5 mm lateral to Bregma and 1.2 ± 0.4 mm posterior to Bregma (mean ± SD, *n* = 4 mice). According to mouse brain atlases, this region is within the whisker primary somatosensory barrel cortex, and we shall henceforth refer to this region as wS1. Computed for this location evoking the largest contralateral retraction in each mouse, the mean change in whisker angle over the 500-ms stimulation period was −14.3 ± 1.2° for the right whisker (i.e. retraction of the contralateral whisker) and 17.0 ± 4.8° for the left whisker (i.e. protraction of the ipsilateral whisker).

Optogenetic stimulation of regions anterior to Bregma evoked protraction of both contralateral and ipsilateral whiskers in each mouse. The stimulus site evoking the largest protraction of the contralateral whisker was located at 0.8 ± 0.7 mm lateral to Bregma and 1.8 ± 0.7 mm anterior to Bregma. This region is typically considered to be part of the primary or secondary motor cortex, and we shall henceforth refer to this region as wM1/wM2. Computed for this anterior location evoking the largest contralateral protraction for each mouse, the mean change in whisker angle over the 500-ms stimulation period was 17.3 ± 5.4° for the right whisker and 17.9 ± 10.4° for the left whisker.

In addition, stimulation of a region posterior to Bregma also evoked protraction of both contralateral and ipsilateral whiskers in each mouse. The stimulus site posterior to Bregma evoking the largest protraction of the contralateral whisker was located at 0.6 ± 0.4 mm lateral to Bregma and 1.2 ± 0.4 mm posterior to Bregma. This region is close to parietal association cortex (PtA), cingulate cortex and retrosplenial cortex, and we shall henceforth refer to this relatively poorly defined region as PtA. Computed for this posterior location evoking the largest contralateral protraction for each mouse, the mean change in whisker angle over the 500-ms stimulation period was 12.7 ± 3.8° for the right whisker and 14.2 ± 3.6° for the left whisker.

We next averaged the whisker motor maps from the four mice to generate a grand-average map of the mean change in whisker angle during the optogenetic stimulus (Fig.2C). This revealed the same overall pattern of evoked movements observed in individual mice, indicating the robustness of the method across different mice. In this average map, the peak of the wS1 retraction region was located at 3.1 mm lateral to Bregma and 1.4 mm posterior to Bregma, with a 13.3 ± 0.8° retraction of the right whisker and 18.3 ± 4.3° protraction of the left whisker. The peak of the wM1/wM2 protraction region in the average map was located at 1.6 mm lateral to Bregma and 2.1 mm anterior to Bregma, with a 16.1 ± 5.1° protraction of the right whisker and 23.0 ± 6.1° protraction of the left whisker. In the average map, the peak of the PtA protraction region was located at 0.8 mm lateral to Bregma and 1.4 mm posterior to Bregma, with a 11.9 ± 3.3° protraction of the right whisker and 14.5 ± 3.4° protraction of the left whisker.

### Mapping the time-dependent change in whisker angle

Whisker movements are highly dynamic and the time-averaged mean change in whisker angle during the optogenetic stimulation could average out important aspects of whisker motor control. The earliest evoked movements are likely to be particularly important to study, since these are likely to reflect the most direct effects of the optogenetic stimulation. We therefore investigated the change in whisker angle in 20-ms bins after the onset of the optogenetic stimulation (Fig.3A). In each mouse, we found that the earliest movements were evoked in the time period 20–40 ms after stimulus onset (Fig.3B). The earliest movements were retraction of the contralateral whisker evoked by stimulation of wS1 (Fig.3B). In the time period 40–60 ms, protraction of both contralateral and ipsilateral whiskers was evident upon stimulation of wM1/wM2. Early bilateral whisker protraction also appeared to be evoked by stimulation of an additional more posterior region, which we labeled PtA. These evoked whisker movements became increasingly large over the first 100 ms of optogenetic stimulation, without an obvious change in the spatial mapping of protraction and retraction movements. Averaging these time-dependent maps across the four mice revealed a robust spatiotemporal organization of the evoked whisker movements (Fig.3C).

### Latency maps for evoked whisker movements

In order to examine when the first evoked whisker movements took place, we measured the time from the onset of optogenetic stimulation until the first time that the whisker angle changed more than ±4° relative to the prestimulus baseline whisker angle for each trial in each mouse (Fig.4A). If the whisker did not change angle by more than 4° then the trial was not included in the analysis. The spatial latency maps of individual mice showed some variability, but in general the shortest latency for evoking movement in each mouse was for the wS1-evoked retraction of the contralateral whisker (Fig.4B), which was also found in the average latency map across mice (Fig.4C). Stimulation of wS1 evoked retraction of the contralateral whisker with a latency of 33.8 ± 21.5 ms and protraction of the ipsilateral whisker with a latency of 61.5 ± 20.4 ms. Stimulation of wM1/wM2 evoked protraction of the contralateral whisker with a latency of 47.8 ± 7.6 ms and protraction of the ipsilateral whisker with a latency of 45.8 ± 7.2 ms. Stimulation of PtA evoked protraction of the contralateral whisker with a latency of 46.5 ± 7.7 ms and protraction of the ipsilateral whisker with a latency of 39.8 ± 8.4 ms.

### Maps of early peak change in whisker angle

In order to further characterize the early-evoked whisker movements, we measured the maximal change in whisker angle during the first 100 ms of stimulation in each trial for each mouse (Fig.5A). In each mouse (Fig.5B) and average across mice (Fig.5C), we observed contralateral whisker retraction (−15.1 ± 2.9°) and ipsilateral whisker protraction (41.9 ± 9.6°) evoked by stimulation of wS1. Stimulation of the wM1/wM2 region evoked bilateral protraction (25.4 ± 9.1° for the right whisker and 32.0 ± 15.5° for the left whisker). The PtA region also evoked bilateral protraction (25.2 ± 4.0° for the right whisker and 25.5 ± 9.3° for the left whisker). In the early peak maps of some individual mice, the anterior wM1/wM2 region appeared relatively localized and separated from the more posteriomedial PtA region (Fig.5B), however, in the average peak maps across mice, the posteriomedial PtA protraction region appeared to be more-or-less continuous with the more anterior wM1/wM2 region (Fig.5C).

### Movement maps for the 5–15 Hz frequency band

Stimulation of some locations evoked rhythmic back-and-forward movements of the whisker (Fig.1C), similar to exploratory whisking, which typically occurs in the 5–15 Hz range. We therefore quantified the amplitude of oscillatory whisker movements by integrating the FFT of the whisker angle across the 5–15 Hz range during the last 400 ms of each optogenetic stimulation trial (Fig.6A). Averaged across trials for each individual mouse (Fig.6B) and in the grand average across mice (Fig.6C), we found that the largest amplitude 5–15-Hz movements of both left (27.5 ± 5.0°) and right whiskers (30.1 ± 3.5°) were evoked by stimulation of PtA. The lowest amplitude 5–15-Hz movements for the contralateral whisker (2.2 ± 0.2°) were evoked by stimulation of wS1. Stimulation of this region however evoked large amplitude 5–15-Hz movements for the ipsilateral whisker (21.8 ± 1.9°). Stimulation of wM1/wM2 also evoked large amplitude 5–15-Hz movements for both left (20.4 ± 3.9°) and right (25.4 ± 6.7°) whiskers.

### Whisker movement maps evoked at lower stimulation intensity

It is likely that lower light powers would stimulate fewer neurons, which might also be more spatially localized. In a subset of sessions, we used a lower mean light power of 0.36 mW (rather than 1.75 mW) to optogenetically evoke movements (Fig.7A). In general, the evoked whisker movements at this lower light intensity were less reliable, delayed and smaller in amplitude. Qualitatively, however, most features of the maps remained unchanged. We compared the grand average maps across mice for the mean change in whisker angle during the optogenetic stimulation (Fig.7B), the latency of evoked whisker movements (Fig.7C), the early peak change in whisker angle over the first 100 ms of stimulation (Fig.7D) and the 5–15-Hz FFT of whisker angle during the last 400 ms of stimulation (Fig.7E). A contralateral whisker retraction region (wS1) was found at both high and low optogenetic stimulus intensities with a similar broad spatial location. The frontal rhythmic protraction region (wM1/wM2) appeared more localized at low stimulus intensities but similarly centered at ∼2 mm anterior and ∼1.5 mm lateral to Bregma. A rhythmic bilateral protraction region located close to the midline and posterior to Bregma (PtA) was also present at low stimulus intensities.

For the mean change in whisker angle during the optogenetic stimulation at low light power (Fig.7B), the wS1-evoked retraction of the contralateral whisker was reduced by 53.6% of the high light power, and the ipsilateral protraction was reduced by 54.8%. The wM1/wM2-evoked protraction of the contralateral whisker was reduced by 64.1% of the high light power, and the ipsilateral protraction was reduced by 78.9%. The PtA-evoked protraction of the contralateral whisker was reduced by 79.9% of the high light power, and the ipsilateral protraction was reduced by 42.4%.

For the latency (Fig.7C), the wS1-evoked retraction of the contralateral whisker was increased by 160.8% of the high light power, and the ipsilateral protraction was increased by 142.8%. The latency of wM1-evoked protraction of the contralateral whisker was increased by 223.8% of the high light power, and the ipsilateral protraction was increased by 266.2%. The latency of PtA-evoked protraction of the contralateral whisker was increased by 101.0% of the high light power, and the ipsilateral protraction was increased by 129.7%.

### Comparison of evoked ipsilateral and contralateral whisker movements

Depending upon the location stimulated, the left and right whiskers could move in either a similar (Fig.1C) or different (Fig.1D) manner (Fig.8A). We first quantified the difference in the evoked change in whisker position by subtracting the angle of the two whiskers (left minus right) (Fig.8B). On average the left whisker protracted more than the right whisker. In many locations there was little difference in whisker angle, suggesting bilaterally symmetric movements. However, stimulation of wS1 and wM1/wM2 evoked an asymmetric whisker movement, with larger protraction of the ipsilateral whisker.

We next correlated the time-dependent angle of the left and right whisker for each trial (Fig.8C). This revealed that whisker movements were in general highly correlated for most locations stimulated, including frontal wM1/wM2 regions and the posteriomedial PtA region. Strongly anticorrelated movement of the left and right whiskers was found only for stimulation of wS1.

### Comparison of sensory and whisker motor maps in mouse dorsal cortex

In order to compare our whisker motor maps with sensory maps of the dorsal mouse cortex we carried out intrinsic optical imaging under anesthesia while delivering deflections of different right whiskers, mechanical tactile tappings of the right forepaw, right hindpaw, tail, lip and tongue, auditory click stimuli and visual stimuli to the right eye (Fig. 9). There was a good match of the locations of the different evoked sensory responses across the four mice (Fig.9A). We next directly compared the contralateral whisker motor map (Fig.2C) with this sensory map (Fig.9B). As expected from mouse brain atlases, there was a clear overlap of the whisker primary somatosensory cortex identified with intrinsic signal optical imaging with the region that drove retraction of the contralateral whisker, which we have consistently labeled wS1. The stripe between wS1 and wM1/wM2 where we observed less optogenetically evoked whisker movements appeared to correspond to the forelimb, hindlimb, tongue and lip somatosensory cortex.

## Discussion

In this study, we delivered blue light stimuli in an unbiased manner to the left dorsal sensorimotor cortex of Thy1-ChR2 mice, and quantified the evoked whisker movements. Whisker movements were evoked by stimulation of many different locations in the dorsal cortex, and whisker motor control may therefore be spatially highly distributed in the mouse neocortex. Stimulation of a region near to wS1 evoked the shortest latency whisker movement, which consisted of a sustained retraction of the contralateral whisker followed by initiation of rhythmic protraction of the ipsilateral whisker. Stimulation of most other regions of the dorsal cortex evoked rhythmic bilateral whisker protraction with slightly longer latencies compared to wS1 and varying amplitudes and degrees of rhythmicity. The regions driving rhythmic whisker protraction might be divided into at least two subdivisions, a frontal region located in the neighborhood of wM1/wM2 and a midline region located near and posterior to Bregma, which we labeled PtA. Below we consider each of these regions separately.

### wS1 evoked retraction of the contralateral whisker

Stimulation of a region ∼3 mm lateral and ∼1.5 mm posterior to Bregma caused retraction of the contralateral whisker (Fig. 2) with short latency (Fig. 3, Fig. 4). This region overlaps with the whisker primary somatosensory barrel cortex wS1 (Fig. 9). Our data are therefore consistent with previous studies suggesting a relatively direct role for whisker sensory cortex in whisker motor control (Matyas et al., 2010, Sreenivasan et al., 2015). Whisker retraction driven by sensory cortex might serve as a negative feedback signal, perhaps serving to attenuate strong sensory input (Matyas et al., 2010).

Layer 5 pyramidal-tract neurons in the barrel cortex project to the spinal trigeminal interpolaris nucleus, which contains many premotor neurons for motor neurons innervating extrinsic muscles (Matyas et al., 2010, Sreenivasan et al., 2015). Extrinsic muscles (i.e. the muscles anchored outside the mystacial pad) pull the whiskers and the mystacial pad in different directions, with prominent retraction caused by contraction of muscles *nasolabialis* and *maxillolabialis* (Dörfl, 1982, Haidarliu et al., 2012, Moore et al., 2013). It is therefore possible that the short latency retraction of the contralateral whisker evoked by stimulation of wS1 is evoked by monosynaptic excitation of premotor neurons in spinal trigeminal nuclei, which in turn innervate motor neurons of the extrinsic muscles. Future experiments must directly test this hypothesized circuit mechanism. It would be of great interest to explore the effects of optogenetically manipulating the relevant neuronal cell-types in different brainstem nuclei connected with the different facial nucleus whisker motor neuron pools. wS1 neurons innervate many other brain regions, and it is likely that they also contribute to the evoked whisker movements.

Interestingly, shortly after the start of contralateral whisker retraction, the ipsilateral whisker begins to protract. The underlying neuronal circuit is unknown, but bilateral connectivity is prominent in cortex, as well as in brainstem and many other motor structures. In head-centered coordinates, the net effect of stimulating the left somatosensory cortex is clockwise rotation of the whiskers, which could be interpreted as a rotation of a ‘foveal’ whisking region to the right. This could represent the beginning of an orienting body movement toward a region of interest from which sensory information is arriving. Here, we only investigated evoked movements in head-restrained mice, and, in future studies, it will be of great interest to carry out the same experiments in freely moving mice, to see if a rightward (clockwise) rotation of the head accompanies the clockwise rotation of the whiskers. It will also be important to study movements evoked by wS1 stimulation in diverse behavioral contexts, such as during the execution of learned tasks, and to examine if stimulation of different specific neuronal cell-types in wS1 evoke different movements.

### Whisker movements evoked by stimulation of frontal cortex

Stimulation of neuronal activity in frontal cortex also evoked whisker movements. Most cortical areas anterior to Bregma evoked bilateral protraction of the C2 whiskers, with latencies slightly longer than the whisker retraction evoked by stimulation of primary sensory cortex. These data are consistent with previous investigations suggesting that stimulation of a frontal region innervated by wS1, located ∼1 mm lateral and ∼1 mm anterior to Bregma (often labeled wM1), evokes prominent contralateral rhythmic whisker protraction (Matyas et al., 2010, Sreenivasan et al., 2015, Sreenivasan et al., 2016). Pyramidal neurons in wM1 send direct projections to brainstem reticular formation, which contains many premotor neurons for motor neurons innervating intrinsic muscle (Matyas et al., 2010, Sreenivasan et al., 2015), as well as a central pattern generator for whisking (Moore et al., 2013, Deschênes et al., 2016). Intrinsic muscles (i.e. the muscles contained within the mystacial pad) attach to the base of individual whisker follicles, and their contraction causes the protraction of that whisker, pivoting the whisker forward around the insertion point in the pad (Dörfl, 1982, Haidarliu et al., 2012, Moore et al., 2013). It is therefore possible that wM1 evokes contralateral whisker protraction by exciting premotor neurons in the brainstem reticular formation, which subsequently excite motor neurons innervating intrinsic muscles driving whisker protraction. Future experiments must carefully test this hypothesis, by optogenetically stimulating and inactivating specific groups of neurons in the brainstem. wM1 also innervates many other brain regions, and it is likely that they will also contribute to controlling whisker movement.

Although stimulating wM1 evoked reliable movements, in our unbiased optogenetic mapping experiments the largest protraction appeared to be evoked by stimulating a region ∼1 mm anterior to wM1. This region may correspond to a premotor-like region of cortex, which could be labeled wM2. M2 regions strongly innervate M1 (Hira et al., 2013, Hooks et al., 2013), and thus stimulation of wM2 could evoke whisker movements by exciting wM1. However, the shortest latency for protraction of the contralateral whisker evoked by stimulation of frontal cortex was typically also located anterior to wM1. It is therefore possible that wM2 has a more direct role in controlling whisker protraction, and future studies must investigate the underlying neuronal circuits linking wM2 to motor neurons innervating the intrinsic muscles driving whisker protraction.

Our results in head-restrained mice differ from a recent study, which concluded that rat “vibrissa motor cortex activity suppresses contralateral whisking” (Ebbesen et al., 2017). There are a number of important differences including species, methods and behavioral context. Further research is necessary to understand the key determinants giving rise to different results.

### Whisker movements evoked by stimulation of posterior midline cortex

Optogenetic stimulation of a midline region close and posterior to Bregma, which we labeled PtA, also evoked strong bilateral whisker protraction. The amplitude of whisker movements in the 5–15-Hz range computed by the FFT of whisker position was larger in this region than for any other cortical region (Fig. 6). Latencies for evoking whisker movements from this posteriomedial region were similar to frontal cortex and slightly slower than wS1-evoked whisker movements (Fig. 4). How activity in this brain region contributes to the control of whisker movements is unknown. This parietal region is an associative area, receiving input from different sensory regions and thus likely integrating multisensory signals. Future studies must investigate how neuronal activity in this region contributes to controlling whisker movements under diverse behavioral conditions.

### Future perspectives

Whisker motor control is complex, with premotor neurons for whisker motor control being widely distributed across brainstem, midbrain and cortex (Hattox et al., 2002, Grinevich et al., 2005, Takatoh et al., 2013, Sreenivasan et al., 2015). Whisker motor control is therefore likely to be regulated by many different synaptic circuits in the rodent brain. There are a large number of limitations to the current study, which need to be overcome by further experimental investigation. It would be of interest to repeat the current experiments at higher spatial resolution, testing different stimulation paradigms, and in mice expressing optogenetic actuators in different cell-types. Optogenetic inactivation maps will also be of obvious importance investigating both the hypothesis that inhibition of some cortical regions might promote whisker movement (Ebbesen et al., 2017), and also the hypothesis that inhibition of some cortical regions, including wS1 and wM1, might reduce the probability for initiation of spontaneous whisking (Sreenivasan et al., 2016). Because optogenetic stimulation and inhibition can evoke changes in activity not just in the photo-stimulated region, but also in axons of passage and downstream synaptically connected brain regions (Otchy et al., 2015, Sreenivasan et al., 2016), it will also be of great importance in future experiments to simultaneously measure the spatiotemporal dynamics of optogenetically evoked brain-wide neural activity. This is now becoming technically possible through combining optogenetic motor mapping with wide-field imaging of cortical activity using fluorescent voltage-sensitive dyes (Grinvald and Hildesheim, 2004, Ferezou et al., 2007, Mohajerani et al., 2013) or genetically encoded activity indicators (Akemann et al., 2010, Minderer et al., 2012, Vanni and Murphy, 2014, Hochbaum et al., 2014, Madisen et al., 2015, Xie et al., 2016, Zhuang et al., 2017).

Our results suggest that a large part of the dorsal cortex of mice can contribute to controlling whisker movement. It is possible that these whisker motor maps can be modulated by behavioral context and learning. It will therefore be important to investigate whisker motor control in different behavioral contexts, measured across learning of simple goal-directed sensorimotor behaviors. Here, we correlated stimulus location with C2 whisker movements, but it is likely that many other movements, such as limb and body movements, would also have been evoked during our experiments, which we did not monitor. Investigating the coordination of diverse types of movements evoked by stimulating a given cortical region may help investigate other potential organizing principles of motor cortex, such as the suggestion of action zones for different types of behavior in primate motor cortex (Graziano et al., 2002).

## Author contributions

M.A. and C.C.H.P. designed the project and wrote the manuscript. M.F.A.P. and C.C.H.P. constructed the experimental setup and carried out pilot experiments. M.A., V.L.R., G.M.C.R. and N.H. carried out all experiments and analyzed data. All authors commented on the manuscript.
